# Ultra-protective ventilation and hypoxemia

**DOI:** 10.1186/s13054-016-1310-9

**Published:** 2016-05-12

**Authors:** Luciano Gattinoni

**Affiliations:** Department of Anesthesiology, Emergency and Intensive Care Medicine, Georg-August-University of Göttingen, Göttingen, Germany

## Abstract

Partial extracorporeal CO_2_ removal allows a decreasing tidal volume without respiratory acidosis in patients with acute respiratory distress syndrome. This, however, may be associated with worsening hypoxemia, due to several mechanisms, such as gravitational and reabsorption atelectasis, due to a decrease in mean airway pressure and a critically low ventilation-perfusion ratio, respectively. In addition, an imbalance between alveolar and artificial lung partial pressures of nitrogen may accelerate the process. Finally, the decrease in the respiratory quotient, leading to unrecognized alveolar hypoxia and monotonous low plateau pressures preventing critical opening, may contribute to hypoxemia.

The capability to remove some of the metabolically produced CO_2_ with artificial lungs using a low extracorporeal blood flow (<1 l/min) and a high extracorporeal gas flow provides a key to significantly reduce mechanical ventilation (the ultra-protective strategy) [1]. This, and the easily established vascular access, make the method attractive for acute respiratory distress syndrome (ARDS) patients, in whom even the standard 6 ml/kg tidal volume (TV) may still produce unacceptable stress and strain [2]. In a recent multicenter study, Fanelli et al. [1] tested the feasibility of the ultra-protective strategy in combination with extracorporeal carbon dioxide removal (ECCO_2_R) in 15 patients with moderate ARDS. The authors concluded that one could use ultra-protective ventilation with a TV of 4 ml/kg without resulting relevant respiratory acidosis. The price of attaining this goal, however, appears quite high. Six of the 15 patients (40 %) with initially moderate ARDS experienced life-threatening hypoxemia and required either extracorporeal membrane oxygenation (ECMO) or the prone position. The authors concluded that the impact of the ultra-protective strategy, ECCO_2_R, on hypoxemia is not clear. We believe, however, that the observed hypoxemia is due to well-defined mechanisms acting simultaneously, which may be summarized as follows:

1. *Gravitational atelectasis*: Whenever ventilation decreases (reduction of TV and/or respiratory rate) the mean airway pressure decreases, and the lungs tend to collapse. The greater the decrease in ventilation and the greater the lung weight, the greater will be the collapse. This phenomenon, albeit to a lesser extent, occurs in normal lungs on induction of anesthesia [3]. This has been observed experimentally during ECCO_2_R and mechanical ventilation [4]. Positive end-expiratory pressure (PEEP) must be increased by an amount sufficient to maintain the same mean airway pressure in order to preserve the open lung volume [5] otherwise some amount of gravitational collapse is unavoidable. In the study of Fanelli et al., the PEEP increase was so low as to be non-significant.
2. *Absorption atelectasis*: At least two conditions favor the appearance of absorption atelectasis. Firstly, when ventilation is very low the amount of oxygen provided to some pulmonary units may be lower than the amount removed by the blood perfusing those units. This produces a collapse of the pulmonary unit when its ventilation (VA)/perfusion (Q) ratio reaches a critical level [6]. Secondly, if the artificial lung is ventilated with a fraction of inspired oxygen (FiO_2_) greater than that used for the natural lung, the partial pressure of nitrogen in the blood perfusing the natural lung is lower than that in the alveoli [7]. Under these conditions the critical VA/Q is reached more rapidly, since not only oxygen but also nitrogen leaves the alveoli. In the study by Fanelli et al., the artificial lungs were ventilated with an FiO_2_ of 1.0 in some of the centers, while the natural lungs were ventilated with an FiO_2_ of 0.5.
3. *Opening pressures*: Sufficient pressure must be applied to reopen the atelectatic areas. Studies show that at least one normal TV every 2 min was mandatory in order to correct or prevent atelectasis, otherwise a consistent reduction in lung volume was observed [4]. This low frequency has an effect similar to sighing [8]. In ARDS patients with a plateau pressure of 25 cmH_2_O, as employed in the study by Fanelli et al., we may estimate that 30 to 40 % of the recruitable lung always remains closed [9, 10]. This implies that oxygenation cannot take place even during inspiration [11], and that newly formed atelectasis cannot be reopened.
4. *Alveolar PaO*_2_*and respiratory quotient*: During ECCO_2_R, the respiratory quotient (R) decreases when the CO_2_ eliminated by the natural lungs decreases. To maintain the same alveolar partial pressure of oxygen (PaO_2_), the FiO_2_ must be increased. This has been proved experimentally [12] and can be easily derived from the alveolar gas equation [13]. The lower the FiO_2_, the greater the impact of R on alveolar PaO_2_. For example, with FiO_2_ = 0.3, partial pressure of CO_2_ = 50 mmHg, and R = 1.0, PaO_2_ is approximately 164 mmHg. With FiO_2_ kept constant at 0.3, alveolar PaO_2_ decreases to 113 mmHg when R is reduced to 0.5 and to as low as 48 mmHg when R is decreased to 0.3. The risk of unrecognized alveolar hypoxia as the cause of hypoxemia must be kept in mind during ECCO_2_R, particularly if a FiO_2_ below 0.4 is being used.

All discussed causes of hypoxemia may be prevented or corrected with the proper use of PEEP, FiO_2_ and recruitment maneuvers, e.g., intermittent sighs.

## Abbreviations

ARDS: acute respiratory distress syndrome
ECCO_2_R: extracorporeal carbon dioxide removal
FiO_2_: fraction of inspired oxygen
PaO_2_: alveolar partial pressure of oxygen
PEEP: positive end-expiratory pressure
Q: perfusion
R: respiratory quotient
TV: tidal volume
VA: ventilation
